# Advances on Neurogastroenterology and Motility Disorders: Pathophysiology, Diagnostics and Management

**DOI:** 10.3390/jcm11102911

**Published:** 2022-05-20

**Authors:** Amir Mari, Edoardo Savarino

**Affiliations:** 1 Gastroenterology Department, Azrieli Faculty of Medicine, Bar Ilan University, Nazareth Hospital, Nazareth 16100, Israel; 2 Department of Surgery, Oncology and Gastroenterology, University of Padua, 31100 Padua, Italy; edoardo.savarino@unipd.it; 3 Gastroenterology Unit, Azienda Ospedale Università di Padova, 35128 Padua, Italy

Symptoms related to abnormalities in gastrointestinal tract motility and functions are very common in the general population, affecting both pediatrics and adults, from both sexes [1]. These symptoms are generally of a chronic nature and may affect the quality of life and any part of the gastrointestinal tract. The symptoms include globus sensation, dysphagia, heartburn and regurgitation, belching, epigastric pain or burning, nausea and vomiting, abdominal pain, bloating, constipation, fecal incontinence and others [1,2,3]. Generally, in organic diseases (inflammatory, autoimmune, neoplastic, etc.), the investigation and management routes are well established and based on known pathophysiological mechanisms and theories. However, this is not the case in functional disorders of the gastrointestinal tract. The general approach consists of excluding organic conditions by performing various blood tests, stool tests, and endoscopy and imaging, especially when alarm signs exist [4]. The majority of the patients, however, have negative investigations despite their debilitating symptoms and impaired quality of life. For these patients physiological testing can help to better understand the origin of the symptoms and naturally improve management.

The last decade has witnessed important advances in diagnostic tools and technologies used for the improved assessment of gastrointestinal function and motility [5,6]. A review by the International Working Group for Disorders of Gastrointestinal Motility and Function revealed that the performance of these diagnostics can identify clinically relevant pathologies that may guide management [7,8]. High resolution manometry (HRM) has become the gold standard modality for the evaluation of esophageal function and has extensively replaced the old conventional systems [9,10]. For patients with dysphagia and esophageal symptoms, the use of HRM systems has enabled a more precise assessment of esophageal and lower esophageal sphincter functions, with an improved ability to localize the lower esophageal sphincter. Importantly, the progress of HRM has enabled the introduction of the Chicago Classification, now at its fourth version, which uses a working scheme dividing esophageal disorders into major and minor esophageal disorders [11]. The Chicago Classification is currently considered the working algorithm for analyzing and interpreting HRM studies. Moreover, the endoluminal functional lumen imaging probe (EndoFLIP) has been developed as a modern technology performed under sedation, used to measure obstructions at the esophageo-gastric junction level by evaluating its distensibility [12]. EndoFLIP may further help to measure secondary peristalsis in patients with esophageal symptoms, enabling the diagnosis of achalasia or other major esophageal motility disorders. Advances in fluoroscopy methodology, such as the timed barium swallow protocol and timed barium surface area measurement, have further added to the diagnostic arsenal when assessing a patient with esophageal symptoms [13,14].

Further research has focused on improving our understanding of GERD pathogenesis, in order to ameliorate our diagnostic approach [15]. Indeed, only 30% of patients with GERD will have a diagnostic endoscopy (i.e., esophagitis or Barrett). The Lyon consensus stated that GERD could be confirmed when acid exposure time is (>6%/24 h) [16]. In patients with an inconclusive diagnosis, novel metrics from impedance-pH monitoring are currently suggested, namely the mean nocturnal baseline impedance (MNBI) and post-reflux swallow-induced peristaltic wave (PSPW) index, which demonstrated the ability to either confirm or refute GERD diagnosis [17]. The advent of wireless pH-monitoring (Bravo capsule) provides a catheter-free approach and enables a prolonged period (up to 96 h) of monitoring, which improves the test’s ability to assess the association of reflux and symptoms and is more easily tolerated by patients compared to the traditional catheter-based systems [18].

Notably, the application of artificial intelligence (AI) to the upper GI tract is mainly in the field of endoscopy; however, AI systems application is expanding in other upper GI settings, to include GERD, eosinophilic esophagitis, and motility disorders [19,20].

As for gastric symptoms, currently, scintigraphy of a solid meal (using a^99m^ Technetium-labeled egg) is considered the gold standard for gastroparesis diagnosis [21]. However, breath tests and wireless motility capsules may be alternatives to scintigraphy for the assessment of gastric emptying [21]. In addition, in this context, EndoFLIP showed some important advancements for the clinically relevant disorders of the pylorus, requiring more invasive therapy when symptoms of gastroparesis are severe [12].

Disorders in the anorectum such as constipation and fecal incontinence are also common in the general population and cause severe impacts on life quality and productivity [22]. High resolution anorectal manometry is a modern tool considered to be the best-established diagnostic tool that permits an objective evaluation of anal and rectal sensory and motor functions. The recent London classification is a practical standardized protocol for the performance and analysis of anorectal manometry [23].

Numerous advances in the field of neurogastroenterology and motility have been achieved in recent years, including new imaging testing, developments at the cellular and molecular levels, the evolving role of the microbiome in various functional gastrointestinal symptoms, the role of diet and traditional and complementary medicine in managing functional gastrointestinal conditions, and many other advances from the clinical and laboratory levels. We believe that this Special Issue, in the Journal of Clinical Medicine, is of paramount significance and relevance for shedding light on the recent advances in neurogastroenterology and motility disorders, from pathophysiology to management, at the clinical and laboratory levels.

## Abbreviations

EndoFLIP	Endolumenal functional lumen imaging probe
GERD	Gastro-esophageal reflux disease
HRM	high-resolution manometry
MNBI	mean nocturnal baseline impedance
PSPW	post-reflux swallow-induced peristaltic wave
AI	Artificial Intelligence
